# INTERPROFESSIONAL TEAM MEMBERS’ PERCEPTIONS OF PALLIATIVE SOCIAL WORKERS’ CONTRIBUTIONS TO PATIENT-CENTERED CARE

**DOI:** 10.1093/geroni/igac059.2528

**Published:** 2022-12-20

**Authors:** Arden O'Donnell, Judith Gonyea

**Affiliations:** Boston University, Medfield, Massachusetts, United States; Boston University, Boston, Massachusetts, United States

## Abstract

Interprofessional collaborative practice (IPCP) is fundamental to the delivery of high-quality patient-centered care. An IPCP core tenet is that efficient and effective teams are crucial for the delivery of care that achieves patient satisfaction as well as positive patient-level outcomes. Although a growing number of IPCP studies explore how IPCP professionals work collaboratively, the vast majority focus on the interactions of physicians and nurses, few consider other professions or simply lump them into an “other” category. This qualitative study addresses this knowledge gap through the exploration of how hospital palliative care teams utilize the skills of advanced hospice and palliative care certified (APHSW-C) social workers to increase teams’ overall effectiveness. A two-step recruitment process was used to create matched samples of 17 palliative social workers and 24 interprofessional team members (i.e., physicians, nurses, physician assistants, chaplains). Using a constructivist grounded theory approach, semi-structured interviews were conducted, recorded, and transcribed. Analysis revealed six themes surrounding other profession's perceptions of social workers’ contributions: 1) providing continuity during and across admissions, 2) assessing and promoting the patient’s bio-psychosocial needs, 3) offering clinical expertise and support to patients with complex psychosocial needs, 4) allocating time to initiate, process and revisit difficult conversations with patients and families, 5) modulating the pace and intensity of emotionally laden conversations, and 6) supporting team well-being. Findings underscore that interprofessional team members view social workers as playing a significant role in developing, building, and sustaining a therapeutic alliance between the patient and the team.

